# Red Mud as an Adsorbent for Hazardous Metal Ions: Trends in Utilization

**DOI:** 10.3390/toxics13020107

**Published:** 2025-01-28

**Authors:** Maja Rajković, Ivana Jelić, Marija Janković, Dragi Antonijević, Marija Šljivić-Ivanović

**Affiliations:** 1 Vinča Institute of Nuclear Sciences, National Institute of the Republic of Serbia, University of Belgrade, 11000 Belgrade, Serbia; ivana.jelic@vin.bg.ac.rs (I.J.); marijam@vin.bg.ac.rs (M.J.); marijasljivic@vin.bg.ac.rs (M.Š.-I.); 2 Faculty of Mechanical Engineering, Innovation Centre, University of Belgrade, 11000 Belgrade, Serbia; dantonijevic@mas.bg.ac.rs

**Keywords:** bauxite residue, adsorption, heavy metals, radionuclides, sustainability

## Abstract

The increasing importance of waste materials utilization with the necessary modification to remove various pollutants from industrial wastewater has been a research focus over the past few decades. Using waste material from one industry to solve pollution problems in another ultimately leads toward sustainable and circular approaches in environmental engineering, solving waste management and wastewater treatment issues simultaneously. In contemporary research and industry, there is a notable trend toward utilizing industrial wastes as precursors for adsorbent formation with a wide application range. In line with this trend, red mud, a byproduct generated during alumina production, is increasingly viewed as a material with the potential for beneficial reuse rather than strictly a waste. One of the potential uses of red mud, due to its specific composition, is in the removal of heavy metal and radionuclide ions. This study summarizes red mud’s potential as an adsorbent for wastewater treatment, emphasizing techno-economic analysis and sorption capacities. An overview of the existing research includes a critical evaluation of the adsorption performance, factors influencing efficiency rather than efficacy, and the potential for specific pollutant adsorption from aqueous solutions. This review provides a new approach to a circular economy implementation in wastewater treatment while guiding future research directions for sustainable and cost-effective solutions.

## 1. Introduction

The trajectory of industrialization has evolved significantly over time. Historically, the primary focus was on economic growth and aligning production with market demands. Today, however, the emphasis has shifted towards achieving sustainable development goals, reflecting a growing awareness of the environmental and health impacts associated with industrial activities.

A critical challenge in this context is the release of heavy metals and radionuclides into the environment, particularly into the biosphere [1,2]. These contaminants pose severe environmental and health risks due to their persistence and potential for bioaccumulation [3]. Heavy metals and radionuclides in wastewater, for instance, not only threaten human health but also degrade the quality of the environment.

Addressing these issues requires innovative strategies for managing industrial waste and mitigating its harmful effects. One promising approach is the transformation of waste materials, such as red mud, into valuable resources [4,5,6]. This strategy not only reduces the environmental footprint of industrial processes but also aligns with the principles of sustainable development by turning waste into a resource.

Over the past ten years, there has been a heightened emphasis on sustainable development, characterized by the responsible utilization of natural and artificial resources, adoption of cleaner energy sources, reduction in industrial emissions, and preservation of the environment. This has led to an increasing focus on repurposing industrial wastes to develop value-added materials that offer environmental and economic benefits.

**Figure 1:**
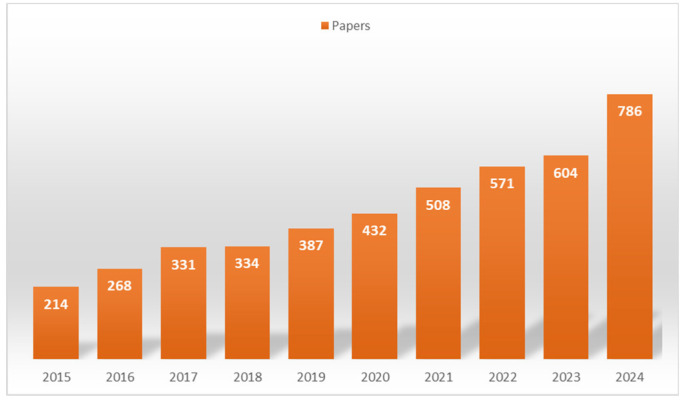
Scientific publications on red mud utilization: a Scopus analysis by year (2015–2025).

Among the industrial wastes being repurposed, red mud stands out as a significant example of an industrial byproduct that has been effectively transformed into valued materials for different applications, thus saving natural resources [7,8,9]. The extensive production and hazardous characteristics of red mud have sparked significant interest in its comprehensive investigations globally [8,10,11]. Figure 1 shows the production of scientific publications dealing with red mud reuse and renewal for different kinds of purposes. This growing body of research highlights the increasing recognition of red mud’s potential across diverse applications, driven by its quantity and potential environmental hazards. The upward trend in publications underscores a global commitment to addressing red mud challenges through innovative and sustainable solutions.

The effective repurposing of red mud addresses the dual challenges of waste disposal and environmental preservation, while also offering opportunities for economic growth. This waste material, a byproduct of bauxite-refining and alumina production processes, is commonly classified into Bayer red mud and Sintering red mud [12,13] depending on the specific process involved. However, its high alkalinity, potential for heavy metal leaching, and radioactivity make its disposal hazardous to the environment.

Currently, the most prevalent management method for this highly alkaline suspension involves landfilling. While this approach is widely used, it occupies large land areas and poses significant environmental risks, including the migration of toxic substances into interconnected water, air, and soil systems. Recognizing these challenges, science and industry are increasingly collaborating to find sustainable solutions for red mud management, aligned with the principles of a circular economy.

**Figure 2:**
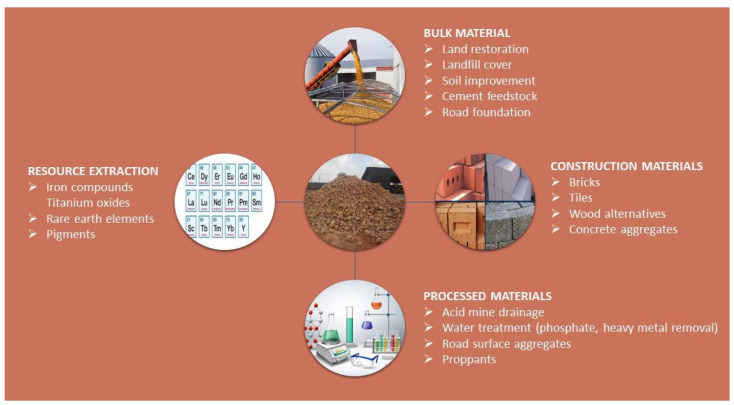
Red mud potential applications.

Valorization efforts have uncovered a range of potential applications for red mud (Figure 2).

These include the recovery of iron, its use in the construction industry for cement production or as a road material, the extraction of rare earth elements, and its application as a catalyst and as an adsorbent to remove various pollutants from water. Specifically, red mud has been shown to be effective in adsorbing heavy metals, radionuclides, phenolic compounds, bacteria, dyes, and acid mine drainage, offering a promising avenue for mitigating environmental pollution while deriving value from this industrial byproduct [9,14,15,16,17,18,19,20,21,22,23].

Red mud is a byproduct derived from the processing of bauxite, predominantly through the Bayer process [24], which is the leading method for producing alumina—a precursor to aluminum production. Annually, the global production of aluminum remains consistent, generating approximately 1.25 tonnes of red mud residue for each tonne of aluminum produced [25]. In November 2024 alone, aluminum production reached 6,040,000 t [26].

According to the European Waste Catalogue (EWC) [27], red mud is classified as mirror hazardous, indicating that it can be either hazardous or non-hazardous depending on specific conditions. This classification permits its application without prior treatment for the remedial purposes of another medium.

While significant progress has been made in understanding the environmental risks and potential applications of red mud [20,28], there is still a need for further development in standardized treatment and utilization methods. Additionally, systematic research on the use of this bauxite residue as an adsorbent for industrial wastewater treatment to remove heavy metals and radionuclides is lacking [29,30,31].

This paper provides a distinctive overview of recent research into the feasibility of using red mud for the removal of heavy metals and radionuclides from wastewater and liquid radioactive waste. In the context of industrial wastewater remediation, removing these pollutants typically requires expensive techniques involving significant capital investment or costly materials such as commercial activated carbon [32]. This situation underscores the need for more economical adsorbents, such as red mud. This study presents a critical analysis of the existing literature regarding the efficacy of red mud in the removal of heavy metals and radionuclides from aqueous solution considering its potential commercialization as a locally available resource. Additionally, the study provides a unique comparative review of red mud adsorption features with the current literature data on other low-cost sorbents based on waste materials.

In summary, the transformation of red mud into a valuable resource exemplifies how industrial byproducts can be repurposed to meet sustainable development goals, thereby addressing environmental challenges and promoting economic growth. Using red mud as a cost-effective adsorbent for the remediation of industrial wastewater not only provides an efficient solution for removing heavy metals and radionuclides but also leverages an otherwise problematic waste material, aligning with both environmental preservation and economic feasibility.

## 2. Properties and Adsorption Potential of Red Mud

### 2.1. Red Mud Characterization

Given the significant differences observed during the analysis of Bayer and Sintering red mud, the description of which is given in the studies of the authors Ping Wang and Dong-Yan Liu [33], this literature review will be oriented to the characteristics of Bayer red mud.

According to data from several studies [33,34,35,36,37], the main chemical composition of red mud is presented in Table 1.

The typical mineral composition, which considers the surrounding conditions of the formation of the red mud, produces a heterogeneous mixture of nanocrystalline particles of metal-oxides and minerals, of which the most significant—Goethite α-FeOOH, Hematite Fe_2_O_3_, Gibbsite Al(OH)_3_, Boehmite α-Al_2_O_3_∙H_2_O, Rutile/Anatase TiO_2_, Quartz SiO_2_, sodalite Na_8_(Al_6_Si_6_O_24_)Cl_2_, cancrinite Na_6_Ca_2_ [(CO_3_)_2_ Al_6_Si_6_O_24_]∙H_2_O, and numerous other minerals in trace amounts—are among the typical mineral assemblage [36,37,38]. However, it is essential to note that the chemical and mineral composition of red mud can differ from one location to another.

### 2.2. Adsorbent Properties

Adsorption has been widely used as an economical, efficient, and environmentally acceptable technique for treating wastewater, being the most cost-effective technology for this purpose [4,5,6,39]. The United States Environmental Protection Agency (US EPA) also recognizes the adsorption process as one of the most effective methods for treating wastewater [40]. This underscores the advantages of adsorption as a remediation mechanism for removing pollutants from wastewater [41]. The adsorption process is particularly effective in removing heavy metal and radionuclide ions due to adsorbents’ large specific surface area, abundance of pores, and potential for chemical reactions, which create a strong attraction for heavy metals and other contaminants. Adsorption has been widely used as an economical, efficient, and environmentally acceptable technique for treating wastewater, being the most cost-effective technology for this purpose.

Previous research on the remediation of wastewater and/or treatment of liquid radioactive waste has encompassed a broad range of potential applications of waste materials as adsorbents for the immobilization of metal ions and radionuclides such as red mud, construction and demolition waste (C&DW), mining tailings, fly ash, blast furnace slag, etc. Thus, there is a noticeable trend in the study of heavy metal and radionuclide sorption that focuses on examining the adsorption characteristics of byproducts and waste materials, particularly end-waste materials. In this context, special attention has been devoted to the possible treatment of diverse wastewaters by red mud, considering that its samples collected worldwide demonstrate excellent removal capacities for various toxic inorganic cations, anions, and organic compounds.

Red mud is recognized as a promising adsorbent due to its unique physicochemical characteristics that make it suitable for use as an adsorbent material in various environmental applications, including wastewater treatment. Except for the mineral composition stated above, some of its key characteristics include high surface area (providing numerous active sites for the adsorption of contaminants), porous nature (allows the easy diffusion of contaminants into its structure), surface functional groups (such as hydroxyl and carboxyl groups, which play a crucial role in the adsorption process), electrostatic interactions (beneficial in the removal of ions from solution), chemical stability, versatility (effective in adsorbing a wide range of pollutants), and potential for its modification and activation and other adsorbent improvement [33,34,35,36,37,38,42,43,44,45,46].

## 3. Literature Overview

The relevant studies on wastewater treatment using red mud are summarized in Table 2, providing a basis for benchmarking and future applications. The table includes a brief overview of modified adsorbents, highlighting their adsorption capacities, adsorbate removal efficiencies, and key adsorbent properties. This compilation is intended to serve as a valuable resource for assessing red mud’s viability as an adsorbent and guides further research and development in this field, aligning with circular economy principles and waste valorization strategies.

**Table 2:** Summary of studies on modified adsorbents: adsorption capacities and key properties for benchmarking and future applications.

A Sample of Red Mud and the Location	Method of Characterization	Adsorption Mechanism	Adsorbent Dose	Applied Adsorbent Modification Method	Pollutant	Adsorption Capacity	Ref.
“Birač” Alumina Factory, Bosnia and Herzegovina	ICP-AES	Inner-sphere complex and/or precipitation/co-precipitation	0.1 g	Dried, powdered raw red mud (without pretreatment)	Co^2+^ (laboratory-prepared solution)	0.52 mmol/g	[46]
		Electrostatic interactions and the ion exchange mechanism			Sr^2+^ (laboratory-prepared solution)	0.31 mmol/g	
		Specific cation adsorption					
	AAS	Inner-sphere complex and/or precipitation/co-precipitation	0.1 g	Rinsed red mud	Co^2+^ (laboratory-prepared solution)	0.203 mmol/g	[47]
		Specific cation adsorption			Sr^2+^ (laboratory-prepared solution)	0.117 mmol/g	
	TGA/DT and AAS	Precipitation of Ni(OH)_2_	5 g/L	Rinsed, heat-treated red mud at 600 °C	Ni^2+^ (laboratory-prepared solution)	27.54 mg/g	[48]
	XRD and AAS	Precipitation of Ni(OH)_2_	5 g/L	Rinsed, heat-treated red mud at 600 °C	Ni^2+^ (laboratory-prepared solution)	0.372 mmol/g	[49]
	AAS, XRD, and FT-IR	Precipitation/co-precipitation	5 g/L	Raw red mud powder	Ni(II) and citrate ions (laboratory-prepared solutions)	27.4 mg/g (1:0) *	[50]
						25 mg/g (1:0.25) *	
						21 mg/g (1:0.5) *	
						7.6 mg/g (1:1) *	
						2.5 mg/g (1:2) *	
	XRD, ATR-FT-IR, and AAS	Precipitation of $Ni{\left(OH\right)}_{2}$	5 g/L	Acid-treated red mud	Ni(II) (laboratory-prepared solution)	11.8 mg/g (0.05 mol/L HCl)	[51]
						9.6 mg/g (0.1 mol/L HCl)	
						1.1 mg/g (0.25 mol/L HCl)	
						1.5 mg/g (1 mol/L HCl)	
	ICP-OES	Co^2+^ inner-sphere complexes and/or the surface precipitates	0.1 g	Rinsed red mud	Co^2+^, Sr^2+^, and Cs^+^ (simulated wastes; laboratory-prepared solutions)	Co^2+^ 0.16–0.44 mmol/g	[52]
		Sr^2+^-specific, with the contribution of ion exchange				Sr^2+^ 0.029–0.19 mmol/g	
		Cs^+^ irreversible fixation and partly ion exchange				Cs^+^ 0.017 mmol/g	
Vedanta Aluminum Industries, Langigarh, Odisha, India	XRD, SEM, EDX, BET, and FT-IR	Metal ion exchange	0.4 g	Thermally activated acid-neutralized red mud (HCl)	Pb(II) (synthetic sample)	6.0273 mg/g	[53]
Alumina factory, north China	XRD, FT-IR, BET, CEC, XPS, and sequential extraction	Metal–metal ion exchange and the specific adsorption (inner-sphere complex formation)	10 g	Heat-treated red mud at 500 °C	Cd(II) (synthetic sample)	42.64 mg/g	[54]
Alumina production plant, southwest China (disposal landfill)	ICP-OES, FT-IR, SEM, and TEM	Physical adsorption and chemical co-precipitation	40 g/L	Non-treated	As(V) (wastewater from non-ferrous smelter)	101.5 mg/g	[55]
Pingguo Aluminum Co.Ltd., Baise city, Guangxi province, China	FT-IR, SEM, and XRD	Physical adsorption	3.0 g/L	Nitric acid-neutralized red mud	U(VI) (standard solution)	29.42 mg/g	[56]
		Endothermic chemical adsorption		Ferric nitrate-modified red mud		32.92 mg/g	
		Physical adsorption		Aluminum nitrate-modified red mud		30.64 mg/g	
	XRD, DTG, SEM, FT-IR, and ICP-MS	Surface electrostatic attraction, physical adsorption by porous structure, and chemical adsorption	3.0 g/L	Carbon-calcined red mud at 600 °C	U(VI) (laboratory-treated natural sample)	48.85 mg/g	[57]
				Iron and carbon-combined calcined red mud at 600 °C		59.45 mg/g	
Shandong Aluminum Industry Company, China	XRF, XRD, BET, XPS, TEM, SEM, TGA, and ICP-AES	Specific adsorption (involves the formation of supported nano zero-valent iron)	0.20 g	Carbothermally treated mixed red mud–coal powder (previous acid-neutralized red mud)	Cr(VI) (synthetic sample)	Not specified	[58]

* Ni/citrate molar ratio. ICP-AES—inductively coupled plasma atomic emission spectrometry; AAS—atomic absorption spectrometer; XRD—X-ray diffraction; SEM—scanning electron microscope; EDX—energy dispersive X-ray; XPS—X-ray photoelectron spectroscopy; BET—Brunauer–Emmett–Teller surface area analyzer; FT-IR—Fourier transform infrared spectroscopy; ATR-FT-IR—attenuated total reflectance Fourier transform infrared spectroscopy; CEC—cation-exchange capacities; TEM—transmission electron microscopy; DTG—thermogravimetric and derivative thermogravimetric; ICP-MS—inductively coupled plasma mass spectrometry; ICP-AES—inductively coupled plasma atomic emission spectrometry; TGA—thermogravimetric analysis; DTA—differential thermal analyses.

### 3.1. Literature Finding Discussion

Through the analysis of the literature, a growing interest over the last decade in the use of red mud as an adsorbent for the treatment of contaminated wastewater can be observed. Particular emphasis has been placed on adsorption studies involving multiple components, as they not only provide insights into the cost-effectiveness of red mud for the removal of heavy metals and radionuclides from wastewater but also ensure a closer approximation to actual wastewater by considering the complex interactions between different pollutants. It is essential to compare the adsorption potential of both individual metals and their combinations, which inevitably interact with each other. This approach offers a more comprehensive basis for optimizing the purification process.

The research by Milenković et al. [46] regarding contact time, pH adjustment, and desorption potential using sequential extraction demonstrated several key findings. Rapid initial adsorption was observed for both cations, followed by a slower rate over time. Although Sr^2^⁺ exhibited faster initial adsorption, Co^2^⁺ showed more effective overall adsorption. The high pH values (≈9.5) were attributed to NaOH from the Bayer process, as the adsorbent was used without prior washing or neutralization. Despite an initial pH increase with Co^2^⁺ and Sr^2^⁺ solutions, the final pH values were significantly lower than in the blanks, converging to equilibrium pH values of approximately 7.2 for both cations. The experimental data conformed well to a pseudo-second-order kinetic model [46]. In the studies of Milenković et al. [46,47], red mud has shown significant potential in the efficient removal of various metal ions simultaneously, indicating its viability as a treatment solution for complex metal-laden wastewater. The continued investigation of these interactions by Milenković et al. [47] has contributed to the consideration of red mud in practical applications, enhancing its effectiveness and sustainability as a wastewater treatment method.

Furthermore, the study by Milenković et al. [47] investigated the adsorption effectiveness and binding mechanisms of Co^2^⁺ and Sr^2^⁺ ions onto rinsed Bosnian red mud under various batch conditions. They varied experimental parameters such as pH, the concentrations of Co^2^⁺ and Sr^2^⁺ in single solutions and binary mixtures, contact time, and the concentration of competing cations and ligands found in liquid radioactive waste. It was observed that Co^2^⁺ adsorption occurred on both positively and negatively charged red mud surfaces, with adsorption increasing as pH rose, showing significant adsorption below the isoelectric point of the rinsed Bosnian red mud at pH 6.6. In contrast, Sr^2^⁺ adsorption increased only on negatively charged surfaces, with marginal adsorption at pH 6.9. The adsorption kinetics followed a pseudo-second-order model, with higher metal concentrations and pH leading to increased adsorption and longer equilibrium times. The rinsed Bosnian red mud showed higher selectivity for Co^2^⁺ compared to Sr^2^⁺, effectively adsorbing Co^2^⁺ even in the presence of other cations, while the adsorption of Sr^2^⁺ was suppressed. In correlation with this, desorption studies revealed a low release of Co^2^⁺ in the presence of competing cations and over the pH range of 3–7, while Sr^2^⁺ is more easily desorbed, especially at low pH and in the presence of Co^2^⁺. A significant finding is that the strong chemical bonds involved in Co^2^⁺ binding, along with the relatively weaker ion exchange and electrostatic interactions for Sr^2^⁺, make washed Bosnian red mud a promising and inexpensive adsorbent for the immobilization of Co^2^⁺ and Sr^2^⁺. This has potential applications in the decontamination of low-level radioactive waste and the remediation of water and soil.

The immobilization of Ni^2+^ ions from aqueous solutions using thermally treated rinsed red mud, as investigated in the study by Smiljanić et al., has proven to be an effective method for nickel removal [48]. This method provides a basis for further research into the application of this adsorbent in wastewater treatment. The adsorbent was prepared by heating rinsed red mud at temperatures ranging from 20 to 1000 °C, with the optimal temperature identified as 600 °C. This thermal treatment enhances the adsorbent’s properties, increasing its capacity to immobilize Ni^2+^ ions from aqueous solutions. Experiments were conducted to optimize the pH value and evaluate the influence of the initial adsorbate concentration on the adsorption efficiency. It was observed that lower pH values significantly enhanced the removal of Ni^2+^ ions. This effect was more pronounced at lower initial concentrations of the adsorbate. Under the optimized experimental conditions, red mud exhibited superior adsorption capacity compared to similar studies. The modified adsorbent achieved an adsorption capacity of 27.54 mg/g, which is approximately 20% higher than the capacities reported in related research under the same set of experimental conditions [49].

Referring to the principles of sustainable development and acknowledging that red mud pretreatment methods such as washing, acid digestion, or annealing can be costly and time-consuming, alongside the preference for an alkaline environment for heavy metal immobilization, Smičiklas et al. [50] investigated the use of raw red mud powder for the removal of Ni(II). Additionally, the study examined the influence of citrate ligands on the immobilization of Ni(II) by red mud, as these ions frequently coexist in various waste streams. The mechanisms and efficiency of Ni^2^⁺ ion removal using untreated red mud were investigated by varying the concentrations of the adsorbent and adsorbate, Ni/citrate ratios, reaction times, and pH values. The results revealed that red mud’s efficacy as an adsorbent medium varies with solution composition, demonstrating the capacity for the sequestration of Ni(II), Ni/citrate complexes, and free citrate anions. However, the presence of citrate induced competitive interactions, resulting in diminished nickel removal due to varying adsorption affinities for the red mud surface. With the increase in the Ni/citrate molar ratio to 1:0, 1:0.25, 1:0.5, 1:1, and 1:2, the maximum adsorption capacities of red mud decreased in the following order: 27.4, 25, 21, 7.6, and 2.5 mg/g, respectively.

Smičiklas et al. also investigated acid treatment on red mud as an adsorbent for Ni(II) adsorption and its stability in the study [51]. The initial adsorbent dose was 5 g/L, while red mud was treated with varying concentrations of HCl (0.05–1 mol/L). The findings revealed that acid treatment significantly altered red mud’s mineralogical composition and surface characteristics. The maximum adsorption capacity for Ni(II) was 11.8 mg/g with 0.05 mol/L HCl-treated red mud, while higher acid concentrations (1 mol/L HCl) led to reduced adsorption efficiency (1.5 mg/g) and stability due to the dissolution of key mineral phases like sodalite and calcite. When compared to raw red mud powder [50], Ni(II) removal by all the treated samples was lower.

An interesting study by Šljivić-Ivanović et al. [52] investigated the potential simultaneous removal of Co^2^⁺, Sr^2^⁺, and Cs⁺ ions from aqueous solutions using economical and abundant adsorbent materials such as washed bauxite residues, calcined bovine bones, and mineral zeolite. What makes this study interesting is precisely the fact that in wastewater treatment, it is often necessary to remove several pollutants simultaneously, which is advantageous for future commercial applications. The researchers explored different approaches to optimize adsorption efficiency, including the use of individual adsorbents, mixtures of adsorbents, and a multi-stage adsorption process, satisfactory results were obtained. The results obtained by Šljivić-Ivanović et al. indicate competitive adsorption between Sr^2^⁺ and Co^2^⁺ on red mud, as discussed in studies [46,47]. Both red mud and calcined bovine bones showed a preference for Co^2^⁺ over Sr^2^⁺ and Cs⁺, whereas mineral zeolite exhibited the highest selectivity for Cs⁺. To increase adsorption capacity, a mixture of adsorbents was tested, varying the order of addition and the mass fraction of each component. The highest total adsorption was achieved with a composite consisting of 20% rinsed red mud, 20% bovine bones, and 60% mineral zeolite, added in that order. The research indicates that by optimizing the process and employing a mixture design methodology for testing the adsorption of multi-component mixtures, a more efficient adsorbent tailored to specific environmental issues can be developed.

To remove Pb(II) from the aqueous solution (a synthetic sample) in batch mode, red mud’s potential as an adsorbent treated with an acid solution was examined in the study by authors Sahu et al. [53]. The intent was to minimize the mud’s high alkalinity and enhance its adsorption characteristics. For this purpose, the efficiency of adsorbent removal was investigated in relation to the initial concentration of lead, solution pH, adsorbent dose, contact time, and temperature. It was determined that a pH of four was optimal and that 6.0273 mg/g was the maximum adsorption capacity. Additionally, the removal of Pb(II) increased as the adsorbent’s initial concentration increased. However, the removal of Pb(II) did not significantly change after 0.4 mg/L, possibly as a result of the overlapping of active sites [53]. A significant amount of Pb(II) was removed (∼88%) in the first 10 min of contact time, keeping the pH and adsorbent concentration at 4 and 0.4 mg/L, respectively. Regarding temperature, the endothermic character of the process was observed [53]. Analyzing initial lead concentrations in relation to possible active sites on the surface of activated red mud is crucial for prospective applications in the removal of Pb(II) from industrial wastewater.

Heat-treated red mud had noticeably improved adsorption characteristics towards Cd(II) according to Yang et al. [54,59]. Examining the impact of heat treatment on the adsorption of hazardous Cd(II) by red mud at temperatures ranging from 200 to 900 °C, the study found that the maximum adsorption capacity 42.64 mg/g and the fastest adsorption rate occurred with heat treatment at 500 °C. A comprehension of the adsorption mechanism is essential due to the heterogeneous composition of heat-treated red mud [49]. Based on XPS analysis and sequential extraction, two mechanisms, namely metal–metal ion exchange and specific adsorption (involving inner-sphere complex formation), were identified as contributing to the adsorption of Cd(II) by heat-treated red mud at 500 °C. Furthermore, the primary mechanism was shown to be specific adsorption. The adsorption capacity is almost doubled compared to raw red mud. In this way, the issue of large amounts of sludge that are deposited in landfills can be solved [54].

The topic of creating artificial adsorbents to remove heavy metals from various media has garnered a lot of attention recently. By comparison with these searches, waste materials such as red mud indicate comparatively good characteristics in terms of adsorbing a range of contaminants from wastewater. According to Lu et al. [55], red mud can be used as an alternative, sustainably acceptable way to effectively remediate high-arsenic wastewater by forming AlAsO_4_@silicate precipitate. It was shown that the highest As removal capacity was at a red mud dosage of 40 g/L, reaching 101.5 mg/g. Lyu et al. and Shabani et al. suggest that the hydroxyl groups in red mud play a key role in capturing arsenate and arsenite, thus increasing their removal efficiency [59,60,61]. A notable observation from the experiments is the importance of modifying the experimental conditions, particularly by adjusting the pH. It is crucial to ensure that the concentration of As in the precipitate does not surpass 5 mg/L. Greater pH values result in decreased leaching of arsenic. In conclusion, this study introduces a cost-effective, easily applicable, and effective approach for treating high-arsenic wastewater. This is attributed to the combination of high adsorption capacity and high arsenic stability due to the creation of alkaline conditions.

Wu et al. showed in their study [56] that raw red mud was effectively modified using nitric acid, ferric nitrate, and aluminum nitrate as neutralization reagents for uranium adsorption. The adsorption capabilities of uranium varied in relation to the initial concentration of uranium (5–200 mg/L) and the applied red mud modification methods. At lower uranium concentrations, nitric acid and ferric nitrate-modified red mud demonstrated greater efficacy in uranium removal compared to red mud modified with aluminum nitrate. It could be concluded that all the modified red muds demonstrated their optimal adsorption capabilities under different uranium concentrations. For nitric acid, aluminum nitrate, and ferric nitrate-modified red mud, the optimal adsorption efficiencies and quantities were 74.50% (1.24 mg/g), 95.56% (12.74 mg/g), and 98.75% (32.92 mg/g), respectively. Additionally, this study indicated that the presence of calcite in red mud reduced the capacity for U adsorption. In correspondence with that, a study by Wu et al. [57] investigated the well-controlled calcination for raw red mud and the optimal U(VI) adsorption capacity of carbon-calcined red mud and iron and carbon-combined calcined red mud. In optimal reaction conditions at a pH of 2.5 and with an initial uranium concentration of 250 mg/L, the adsorption capacity of iron and carbon-combined calcined red mud reached 59.45 mg/g, whereas carbon-calcined red mud exhibited a lower capacity of only 48.85 mg/g (under 600 °C calcination temperature). In conclusion, the practicality and flexibility of this red mud modification method for commercial applications are enhanced by employing a low calcination temperature (600 °C) and cost-effective, readily available additives, such as a carbon and iron mixture. While the study indicated a decrease in adsorption ratios in uranium drainages compared to uranium solution concentrations, possibly influenced by coexisting ions such as Ca, Si, K, and Na, the findings imply that specifically iron and carbon-combined calcined red mud holds promise as an effective material for U(VI) adsorption from radioactive wastewater.

In study [58], Li et al. demonstrated an innovative method of red mud modification that was investigated to enhance its Cr(VI) adsorption characteristics. Through the carbothermal treatment of a mixed powder of red mud and brown coal, an affordable adsorbent material with dispersed nano zero-valent iron was produced. The study demonstrated that the formation of supported nano zero-valent iron provides highly active sites for Cr(VI) removal. This indicates that the nano zero-valent iron component plays a key role in separating Cr(VI) from water and depositing it onto the modified adsorbent. During the tests, it was noted that the modified medium exhibited an efficiency of 53%, categorizing it as having moderate effectiveness. However, what stands out is the intriguing observation that the efficiency of Cr(VI) adsorption progressively rose with the number of cycles. By the fifth cycle, the efficiency approached 100%. In conclusion, the study highlights the successful immobilization and gradual accumulation of collected chromium species through the formation of a highly stable chromite phase (FeCr_2_O_4_) during the carbothermal regeneration process of the spent adsorbent. This paves the way for prospective practical applications and environmentally sustainable solutions in waste streams containing chromium.

### 3.2. Red Mud Adsorption Features vs. Other Waste Materials

To adequately assess the potential of the studied waste material, i.e., red mud through the literature, it is necessary to consider the adsorption characteristics of various waste materials in terms of their capacity to remove heavy metals and radionuclides from aqueous solutions. In order to compare the adsorption efficiency of various investigated waste-based adsorbents from earlier studies with red mud samples, two tables are provided. Table 3 shows the raw and various modified red mud adsorbent comparison. Table 4 displays some other investigated waste-based adsorbents. By comparing the data from Table 3 and Table 4, it is possible to determine the efficiency of red mud in relation to other waste-based adsorbents.

**Table 3:** Modified red mud adsorbents: a comparative analysis.

Adsorbent	Target Pollutant	Adsorption Capacity, mmol/g	Main Findings	Ref.
Raw red mud	Co^2^⁺	0.520	Potential in the efficient removal of Co^2^⁺ and Sr^2^⁺ simultaneously.	[46]
	Sr^2^⁺	0.310		
	Cd^2^⁺	0.286	The adsorption rate of untreated red mud was approximately four times slower compared to that of red mud subjected to thermal treatment at 500 °C.	[54]
Rinsed red mud	Co^2^⁺	0.510	An increase in the initial pH and metal concentration resulted in higher sorption capacities. For the most diluted solution with an initial pH of 5, equilibrium for both cations was achieved almost immediately.	[47]
	Sr^2^⁺	0.205		
	Ni^2^⁺	0.372	Prove to be an economical, composite adsorbent for aqueous Ni^2+^ ions.	[49]
	Co^2^⁺	0.160–0.440	The dominant interaction between the cations resulted in diminished Sr^2^⁺ sorption in the presence of Co^2^⁺.	[52]
	Sr⁺	0.029–0.190		
	Cs⁺	0.017		
Thermally treated red mud	Ni^2^⁺	0.470	Improvement by annealing raw red mud powder at the optimum heating temperature of 600 °C, leading to improved adsorption efficiency.	[49]
	Cd^2^⁺	0.379	Thermal treatment at 500 °C yielded the highest sorption capacity, as this temperature notably enhanced both the specific surface area and the maximum sorption capacity.	[54]
Acid-treated red mud	Ni^2^⁺	0.019–0.201	Findings revealed that acid treatment significantly altered red mud’s mineralogical composition and surface characteristics (higher acid concentrations led to reduced adsorption efficiency).	[54]
	Pb^2^⁺	≈0.030	The removal efficiency gradually increased as the pH decreased, with the highest removal observed at pH 4.	[53]
Hydrothermally modified red mud	Pb^2^⁺	2.662	The modification using colloidal silica and NaOH led to the formation of a zeolite structure, resulting in a significant enhancement of adsorption capacity, increasing it several times.	[59]

Table 4 lists zeolite and hydroxyapatite, widely recognized as effective sorbent materials with high capacities for removing toxic heavy metals [52,62,63,64,65], but also waste-based adsorbents such as calcined beef bones [54,66,67], concrete [68,69,70,71], bricks [69,70,71], and ceramic and roof tiles [70,72].

Combining these wastes, as mentioned in the work by Šljivić-Ivanović et al. [35], along with optimizing the parameters and conditions, can provide key guidelines for utilizing these waste materials for environmental remediation.

**Table 4:** The various waste-based adsorbents comparison.

Adsorbent	Target Pollutant	Adsorption Capacity, mmol/g	Main Findings	Ref.
Calcined bovine bones	Co^2^⁺	0.140–0.460	Co^2+^ adsorbed adequately in the presence of Sr^2+^ and Cs^+^ ions; removal of Sr^2+^ was more suppressed in the presence of Co^2+^ than Cs^+^ ions; for Cs^+^ ions, the assessment of the influence of coexisting cations is impractical.	[52]
	Sr⁺	0.070–0.310		
	Cs⁺	0–0.030		
Waste concretes	Sr⁺	0.250	The results revealed that C&DW has the potential to separate metal pollutants from aqueous solutions. Findings highlight the potential of cement-based materials in treating and conditioning radioactive waste due to their high adsorption capacity.	[68,69]
	Co^2^	0.270–0.320		
	Ni^2^⁺	0.130–0.540		
Waste bricks	Sr⁺	0.010–0.050		
	Co^2^	0.030–0.060		
	Ni^2^⁺	0.130–0.170		
Facade material	Sr⁺	0.250		
	Co^2^⁺	0.120		
	Ni^2^⁺	0.300		
Waste asphalt	Sr⁺	0.020		
	Co^2^⁺	0.060		
	Ni^2^⁺	0.130		
Ceramic tiles	Sr⁺	0.030	Based on the predicted adsorption capacities of metals from a multi-component solution, and considering the generated amounts of this waste, it is assumed that it can be used in the treatment of wastewater.	[72]
	Co^2^⁺	0.170		
	Ni^2^⁺	0.120		
Roof tiles	Sr⁺	0.030	Similar potential for wastewater treatment is assumed for roof tiles.	[72]
	Co^2^⁺	0.060		
	Ni^2^⁺	0.100		
Zeolite	Co^2^⁺	0.013–0.056	Co^2+^ removal was suppressed in the presence of other ions in multi-component mixtures; least selective towards Sr^2+^ and best adsorbent for Cs^+^ ions.	[55]
	Sr^2+^	0.049–0.140		
	Cs^+^	0.140–0.680		
Copper slag flotation tailings (CSFT)	Cd(II)	0.081	Limited effectiveness in removing metal ions from aqueous solutions (sorption capacities decreased in the sequence Cd(II) > Pb(II) > Zn(II) > Mn(II) > Ni(II) > Co(II)); potential as a low-cost and easily accessible sorbent.	[73]
	Pb(II)	0.035		
	Zn(II)	0.032		
	Mn(II)	0.029		
	Ni(II)	0.022		
	Co(II)	0.012		

Red mud showed significant potential as a cost-effective adsorbent for the removal of heavy metals and radionuclides, such as Co^2^⁺, Sr^2^⁺, and Ni^2^⁺, from water, with the highest adsorption capacity of 0.52 mmol/g for Co^2^⁺ [46]. Supporting sustainability, raw red mud demonstrated the highest adsorption capacity. Waste adsorbents showed varying degrees of effectiveness in removing heavy metals and radionuclides. Calcined bovine bones, enhanced by thermal treatment, exhibited a high adsorption capacity of 0.46 mmol/g for Co^2^⁺ [36], indicating their potential for effective radionuclide removal. Šljivić-Ivanović et al. [68] and Jelić et al. [69] investigated the various composite compounds of C&DW and the results showed that waste concretes and facade material demonstrate the high adsorption capacities, particularly for the removal of Ni^2^⁺. Taking into account chemical compatibility with mixtures commonly used for the immobilization of radionuclides and proven high affinity for the studied cations, these waste adsorbent materials could be very effective for the treatment and conditioning of radioactive waste and/or treatment of industrial wastewater. By reviewing the literature, it was determined that the adsorption capacities of metals from multi-component solutions indicate that both red mud and calcined bovine bones can be used in wastewater treatment. Zeolite demonstrates good adsorption characteristics for many metal ions due to its specific porous structure, but it has a low adsorption capacity for some ions, such as Ni^2^⁺ and Cr^3^⁺. To improve its characteristics, Sokić et al. [74] synthesized a composite based on zeolite and hydroxyapatite, which exhibits a significantly higher adsorption capacity compared to natural zeolite. Mineral zeolite from Vranjska Banja [36] has been shown to be the most effective adsorbent for removing Cs⁺ ions, while the removal of Co^2^⁺ was suppressed in the presence of other ions in multi-component mixtures. By reviewing the available literature, it is evident that multi-component adsorption studies are crucial for evaluating the applicability of red mud as a cost-effective adsorbent for the removal of heavy metals and radionuclides from wastewater. It is necessary to compare the adsorption possibilities of both individual metals and their combinations, which inevitably interact with each other, and to optimize the process accordingly. Although some studies indicate the effectiveness of composite materials with magnetic nanoparticles, the study by Dimović et al. [73] demonstrates inferior sorption results compared to red mud in terms of removing metal ions from aqueous solutions. These findings emphasize the importance of considering the specific composition of wastewater and the selective adsorption capacity of various waste materials when designing a treatment system.

### 3.3. Geographical Spread

**Figure 3:**
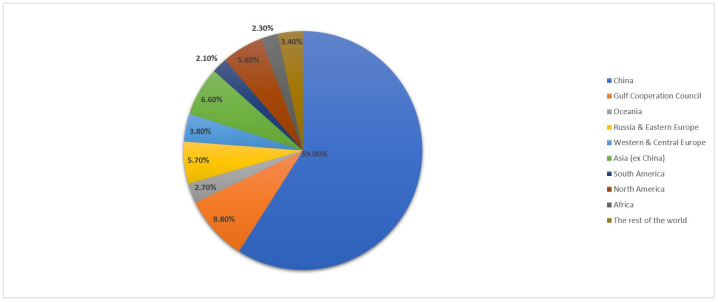
A schematic representation illustrating the global-level spatial distribution of primary aluminum production.

The term “red mud” encompasses a variety of chemical and mineralogical compositions, which can vary significantly depending on the sources of bauxite ores and the refining processes applied. This variability underscores the importance of considering the local conditions and production methods when studying or handling red mud [75]. According to statistics from the International Aluminum Institute, it can be concluded that China maintains an absolute monopoly in the spatial distribution of primary aluminum production from bauxite ore with a total global share of 59% [3,57,58]. This geographical concentration aligns with the substantial quantities of red mud generated in the process. Consequently, it becomes evident why the majority of research efforts related to the utilization of red mud originate from this region. Figure 3 displays a schematic representation of the distribution.

**Figure 4:**
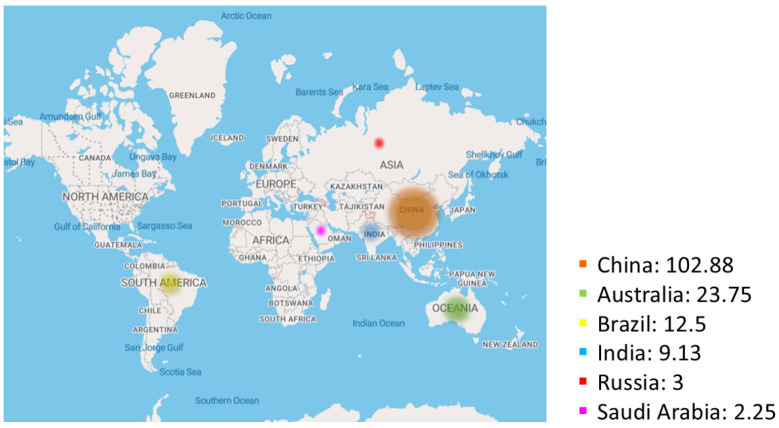
Worldwide spatial distribution of deposited red mud (in millions of tonnes).

Figure 4 provides a distribution of deposited red mud worldwide. In 2023, global aluminum refineries were theoretically estimated to have generated about 177.25 million tonnes of red mud [25,26] based on 1.25 tonnes of red mud generated for each tonne of aluminum.

**Figure 5:**
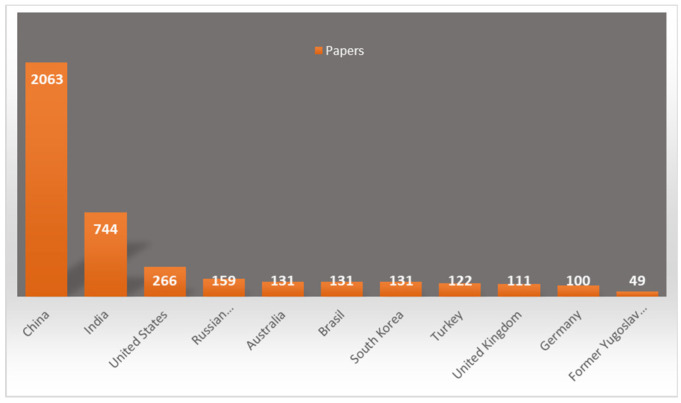
Global distribution of scientific publications on red mud utilization by country: a Scopus analysis (2015–2025).

In accordance with geographical availability and the principles mentioned, the contemporary focus on sustainable development emphasizes the rational use of natural and artificial resources. Utilizing waste materials as valuable resources not only supports this goal but also provides local availability and economic feasibility compared to conventional adsorbents. This trend is evident in the regional distribution of scientific publications presented in Figure 5, which illustrates how research efforts align with the availability of red mud in different parts of the world. The data illustrate how the availability of this industrial byproduct in specific regions influences its investigation and utilization.

In this context, the local availability of red mud, a byproduct of the Birač Alumina factory, offers a compelling justification for its commercial application. All the former Yugoslav republics can benefit from the cost-effectiveness of using red mud as an adsorbent, aligning with the principles of sustainable development and the efficient use of resources.

## 4. Bauxite Residue Applications in Wastewater Treatment

### 4.1. Recent Trends

The current literature often focuses on the general properties [76,77] and potential uses of red mud [20,78], but there is a shortage of detailed, localized studies that consider the specific chemical compositions resulting from different bauxite sources and processing techniques. Such studies are crucial for developing the adsorbent that can compete with conventional adsorbents and find commercial applications.

Contemporary studies addressing bauxite residue, often considered a resource with limited utilization rather than a waste material, emphasize the importance of applying sustainable development concepts to enhance the reuse and potential commercialization of bauxite residue as an adsorbent for wastewater treatment. However, nearly all research indicates the necessity of scale-up, highlighting the need to verify the laboratory-derived results to assess the reaction and kinetic processes under real-world conditions. Gauthier et al. [79] demonstrated that Venezuelan red bauxite residue shows significant potential for remediating waters with acidic mine drainage (pH 4–5), with the ability to adsorb As, Pb, and other inorganic pollutants such as Zn and SO_4_^2−^. This research should be expanded by combining the experimental results with numerical modeling to predict the long-term behavior of this adsorbent in industrial applications. Forghani Tehrani et al. [80] investigated using neutralized red mud to remove Pb(II) from wastewater in the battery industry. The obtained adsorption efficiency of 91% for Pb(II) removal, with an output pH of the treated wastewater at 6.2, which is compliant with Iranian regulatory frameworks, and the identification of optimal conditions regarding the adsorbent amount and contact time, provides strong indications for its potential use in industrial processes. However, despite the treated wastewater meeting regulatory standards, additional studies will be needed to confirm the long-term sustainability of this process in different industrial environments. Regarding the treatment of radioactive wastewater, red mud also demonstrates promising properties. Chen et al. [81] investigated the efficiency of red mud as an adsorbent and found that red mud particles smaller than 5 μm exhibited the highest adsorption capacity for U(VI) from simulated radioactive wastewater. Adsorption based on chemisorption, combined with the stability afforded by mineral phases such as cancrinite, katoite, grossular, calcite, and calcium-aluminosilicates, and the presence of iron-bearing minerals contributing to redox precipitation and further U(VI) adsorption suggests that red mud holds potential for cleaning uranium-contaminated sites and indicates the need for the development of more efficient technologies for radioactive waste treatment. Bai et al. [82] studied the competition in removing metal ions from wastewater. A significant challenge during the simultaneous adsorption of Pb, Cd, and Cu was that higher concentrations of heavy metal ions increased the competitive effect, leading to a reduction in removal efficiency. To address these challenges, they optimized process parameters by considering the effects of temperature and nonlinear adsorption and desorption processes. This study provides valuable insights into the use of industrial red mud as an effective adsorbent for the removal of heavy metals from aqueous solutions. Other studies also address this topic, including the formation of new materials through modification techniques. One such study by Zhao et al. [83] described the synthesis of hydrogels based on red mud, polyacrylic acid, and sodium carboxymethyl cellulose using free radical polymerization. The study suggests that the hydrogel provides promising results for removing heavy metals from contaminated waters, highlighting red mud as a valuable material for addressing heavy metal contamination. However, the research also highlights challenges in multi-component systems containing multiple heavy metals. The adsorbent demonstrated a higher adsorption capacity for Pb(II) compared to Cu(II) and Cd(II). With appropriate optimization of parameters, the application of hydrogels for the effective removal of heavy metals from wastewater in industrial settings will be feasible.

When it comes to the existing review papers on the utilization of red mud as an adsorbent for heavy metals and radionuclides in wastewater treatment, they underscore its effectiveness and ecological benefits as a remediation agent. However, they place less emphasis on practical applications in wastewater treatment and the commercialization of this readily available secondary raw material. Table 5 illustrates a brief overview of review papers from the past five years, addressing the application of red mud as an adsorbent for heavy metals and radionuclides in wastewater treatment. These reviews highlight the material’s effectiveness and ecological benefits but often lack emphasis on its practical application and commercialization.

To increase the adsorption efficiency and overall utility of red mud as an adsorbent, future research should focus on the challenges associated with the fine particle nature of red mud-based adsorbents, which limits their recyclability and reuse. Additionally, improving the recovery efficiency of these adsorbents remains a critical area for further development. By tackling these issues, it will be possible to enhance both the environmental and economic viability of red mud in wastewater treatment applications. However, it is important to note that the current and future trends in this area require a more comprehensive approach, which would include not only technological innovations but also regulatory and economic aspects in order to ensure the long-term sustainability and efficiency of using red mud as an adsorbent.

A proposal for further research in this area matches the need for collaboration between science and industry to remove heavy metals and radionuclides from industrial wastewater in this region. This would involve utilizing this specific adsorbent with appropriate modifications. Additionally, benchmarking and a structured approach to designing a distributed wastewater treatment system [76] would be necessary to assess performance and effectiveness.

### 4.2. The Drawbacks and Advantages

One of the specific advantages of sustainable and circular approaches to wastewater treatment is the use of end-of-life materials with good characteristics that are readily available in large quantities, such as red mud [25,26]. This can reduce procurement costs compared to commercial adsorbents. However, its high alkalinity, potential for heavy metal leaching, and radioactivity often make its practical use challenging. Previous research on wastewater remediation indicates advantages in terms of the versatile adsorption capacity of a wide range of pollutants, including heavy metals and radionuclides, even in complex mixtures and at low concentrations. Additionally, high adsorption capacity at different pH values has been demonstrated, with the possibility of adapting to various conditions (e.g., thermal treatment or washing).

If necessary, red mud can be modified (e.g., by acid treatment, washing, heat, or adding additives) [53,54,55,56] to improve sorption efficiency. However, such treatments, to ensure the future safety of the bauxite residue usage, pose the greatest challenges during real applications. It should be noted that these additional processing steps require additional resources, which increases both costs and process complexity.

Further, cited as an advantage is the fact that using waste materials such as red mud contributes to the reduction in waste in landfills and enhances the sustainability of the overall water purification process. When considering the justification of using red mud in wastewater treatment, the chemical stability of the adsorbent itself should also be taken into account. Some modifications, such as binding with nanoparticles (e.g., zero-valent iron) [58], increase the stability of pollutants and allow for their safe immobilization, while some pollutants (e.g., Sr^2^⁺) [47] can easily desorb under certain conditions, which can lead to secondary contamination. Therefore, in all adsorption-based water purification systems, it is crucial to examine the desorption process, which is particularly important when assessing feasibility at an industrial level.

Excessively aggressive modifications (e.g., treatment with high concentrations of HCl) [51] can cause the dissolution of key mineral phases (e.g., sodalite and calcite), reducing both efficiency and stability of the adsorbent. However, its application in multiple cycles [58], where the adsorption capacity increases, has proven to be especially important for industrial use.

When it comes to the removal of metal ions, the selectivity of adsorption must also be considered, as some metals (e.g., Sr^2^⁺) show a weaker adsorption capacity compared to others (e.g., Co^2^⁺) [47], which can limit efficiency in multi-component systems.

Although challenges remain in fully industrializing this type of adsorbent, given the large number of research that has confirmed its good characteristics in removing heavy metals and radionuclides [46,47,52,56,57,59], while also noting the variations in the composition of bauxite residue depending on the source of the bauxite ore, it is still important to consider the use of this otherwise waste material. It represents an efficient, economically viable, and sustainable resource. With the growing projections of bauxite residue, the long-term application and commercialization of red mud as an adsorbent can significantly contribute to sustainable waste management and effective environmental protection.

## 5. Concluding Remarks

In the remediation of wastewater from diverse industrial sources, the removal of pollutants such as heavy metals and radionuclides often necessitates the implementation of cost-intensive techniques, characterized by either substantial capital investments or the procurement of expensive materials like commercial activated carbon. Industries that generate significant amounts of red mud, such as alumina refining, may find cost-effective applications in meeting discharge limits related to heavy metals and pH regulation, as well as the regulation of radionuclides through the suitable modification of the raw adsorbent. Categories of industrial wastewater that can contain these contaminants, requiring necessary treatment, typically encompass the metal processing and mineral industry, chemical and mineral industry, and wastewater stemming from Nuclear Power Plants.

The hazardous nature of heavy metals and radionuclides to both human health and the environment results from their toxicity and persistence in the environment. Moreover, the process of biomagnification within the food chain has been identified. While regulatory limits for heavy metals and radionuclides in industrial wastewater can vary by country and region, there are general norms (typical ranges of values) for the release of these contaminants into natural recipients after the treatment of industrial wastewater. The emission limit values depend on the production process, the characteristics of the wastewater, and the applied treatment, as well as on the ecological and chemical potential of the recipient. For each specific case, the competent authority determines the emission limit values for discharge.

In each of the aforementioned studies, red mud demonstrated potential and effectiveness as an adsorbent, exhibiting an affinity for removing various pollutants from industrial wastewater through suitable modifications. It functions as an adsorbent for the successful removal of heavy metal pollution from aqueous solutions, along with radionuclides, making it a cost-effective and economically viable option for wastewater remediation.

## Figures and Tables

**Table 1:** The main chemical composition of red mud with their approximate mass percentages.

Component	Content, wt.%
Fe_2_O_3_	20–60
Al_2_O_3_	10–30
SiO_2_	2–20
CaO	2–8
TiO_2_	Trace–28
Na_2_O	2–10

**Table 5:** Recent review papers on the application of red mud as an adsorbent for heavy metals and radionuclides in wastewater treatment.

Keywords	Main Findings	Reference
industrial by-products; composite; adsorption; heavy metal; wastewater purification	Explores industrial wastes (slag, sludge, red mud, lignin, and fly ash) as economical and effective adsorbents for heavy metal removal from wastewater.	[84]
red mud; environmental remediation; polluted water; waste gas; soil	Covers the background, properties, and applications of red mud as an adsorbent, systematically comparing methods for removing metal and non-metal elements from wastewater, with a focus on surface modification.	[85]
adsorption; wastewater treatment; waste gas purification; soil remediation; RM-ERMs	Reviews methods utilizing red mud for environmental remediation, focusing on its application in treating waste streams, including wastewater treatment and heavy metal adsorption.	[4]
red mud; cadmium; contaminant immobilization; environmental remediation; heavy metal sorption	Assesses the potential of red mud for cadmium removal from soil and water, suggesting its prospective use in engineered wastewater treatment systems.	[86]
alumina refining; caustic material; waste treatment; waste valorization; research trend	Analysis of the red mud literature highlights its potential for remediation, focusing on harmfulness minimization and wastewater treatment, with key findings on characterization, treatment methods, metal recovery, environmental applications, and construction uses.	[87]

## Data Availability

Not applicable.

## References

[1] Kampa M., Castanas E. (2008). Human health effects of air pollution. Environ. Pollut..

[2] Nies D., Silver S. (2007). Molecular Microbiology of Heavy Metals.

[3] Nieboer E., Richardson D.H. (1980). The replacement of the nondescript term ‘heavy metals’ by a biologically and chemically significant classification of metal ions. Environ. Pollut. Ser. B Chem. Phys..

[4] Wang M., Liu X. (2021). Applications of red mud as an environmental remediation material: A review. J. Hazard. Mater..

[5] Li X.G., Huang M.R., Wang H.Y., Peng Q.Y., Li X.G., Huang M.R. (2024). Strong Adsorbents for Heavy and Noble-Metal Ions. Milestones in Powerful Adsorbents of Heavy-Metal Ions.

[6] Yamashita K., Kurita K., Ohara K., Tamura K., Nango M., Tsuda K. (1996). Syntheses of thiacrown ethers polymers and their application for heavy metal ion adsorbents. React. Funct. Polym..

[7] Smičiklas I., Jović M., Janković M., Smiljanić S., Onjia A. (2021). Environmental safety aspects of solid residues resulting from acid mine drainage neutralization with fresh and aged red mud. Water Air Soil. Pollut..

[8] Mi H., Yi L., Wu Q., Xia J., Zhang B. (2022). A review of comprehensive utilization of red mud. Waste Manag. Res..

[9] Wang S., Ang H.M., Tadé M.O. (2008). Novel applications of red mud as coagulant, adsorbent and catalyst for environmentally benign processes. Chemosphere.

[10] Sutar H., Chandra Mishra S., Kumar Sahoo S., Prasad Chakraverty A., Sekhar Maharana H. (2014). Progress of Red Mud Utilization: An Overview. Chem. Sci. Int. J..

[11] Liu Y., Naidu R., Ming H. (2011). Red mud as an amendment for pollutants in solid and liquid phases. Geoderma.

[12] Altenpohl D.G. (1980). Materials in World Perspective.

[13] Askeland D.R., Askeland D.R., Fulay P.P., Wright W.J. (1996). Polymers. The Science and Engineering of Materials.

[14] Archambo M.S., Kawatra S.K. (2020). Utilization of Bauxite Residue: Recovering Iron Values Using the Iron Nugget Process. Miner. Process. Extr. Metall. Rev..

[15] Li X.-F., Zhang T.-A., Lv G.-Z., Wang K., Wang S. (2023). Summary of Research Progress on Metallurgical Utilization Technology of Red Mud. Minerals.

[16] Akcil A., Swami K.R., Gardas R.L., Hazrati E., Dembele S. (2024). Overview on Hydrometallurgical Recovery of Rare-Earth Metals from Red Mud. Minerals.

[17] Bhatnagar A., Vilar V.J.P., Botelho C.M.S., Boaventura R.A.R. (2011). A review of the use of red mud as adsorbent for the removal of toxic pollutants from water and wastewater. Environ. Technol..

[18] Vuković J., Perušić M., Stopić S., Kostić D., Smiljanić S., Filipović R., Damjanović V. (2024). A review of the red mud utilization possibilities. Ovidius Univ. Ann. Chem..

[19] Samal S. (2021). Utilization of Red Mud as a Source for Metal Ions—A Review. Materials.

[20] Taneez M., Hurel C. (2019). A review on the potential uses of red mud as amendment for pollution control in environmental media. Environ. Sci. Pollut. Res..

[21] Saveliev S.G., Yarosh T.P., Kondratenko M.M., Babaievska O.V., Baboshko D.Y. (2024). Current state and prospects of red mud utilisation: A review. Earth Environ. Sci..

[22] Cui W., Cui Q., Dong X., Liu J., Song K., Xie M., Yao X. (2024). Current research status and emerging trends in utilization of red mud resources: A study based on bibliometric network analysis. Constr. Build. Mater..

[23] Zhang J., Yao Z., Wang K., Wang F., Jiang H., Liang M., Wei J., Airey G. (2021). Sustainable utilization of bauxite residue (Red Mud) as a road material in pavements: A critical review. Constr. Build. Mater..

[24] Hind A.R., Bhargava S.K., Grocott S.C. (1999). The surface chemistry of Bayer process solids: A review. Colloids Surf. A Physicochem. Eng. Asp..

[25] Shanghai Metals Market (SMM)—European Waste Catalogue. https://news.metal.com/newscontent/103082883/Red-mud-generation-trend-across-major-countries,-2018-to-2023/.

[26] International Aluminium Institute—Primary Aluminium Production. https://international-aluminium.org/statistics/primary-aluminium-production.

[27] Your Dsposal—European Waste Catalogue. https://dsposal.uk/ewc-codes/01/01-03/01-03-10star.

[28] Mintaș O.S., Simeanu C., Berchez O., Marele D.C., Osiceanu A.G., Rusu T. (2023). Impact of Red Sludge Dumps, Originating from Industrial Activity, on the Soil and Underground Water. Water.

[29] Wang S., Jin H., Deng Y., Xiao Y. (2021). Comprehensive utilization status of red mud in China: A critical review. J. Clean. Prod..

[30] Dimović S., Šljivić-Ivanović M., Jelić I. (2019). Utilization of waste materials in heavy metals and radionuclides imobilization by sorption. Tehnika.

[31] Smičiklas I., Jović M., Šljivić-Ivanović M., Milenković A., Smiljanić S. Metals speciation in bauxite residue with implications to its use as an immobilisation agent. Proceedings of the Bauxite Residue Valorisation and Best Practices Conference.

[32] Chai W.S., Cheun J.Y., Kumar P.S., Mubashir M., Majeed Z., Banat F., Ho S.H., Show P.L. (2021). A review on conventional and novel materials towards heavy metal adsorption in wastewater treatment application. J. Clean. Prod..

[33] Wang P., Liu D.Y. (2012). Physical and Chemical Properties of Sintering Red Mud and Bayer Red Mud and the Implications for Beneficial Utilization. Materials.

[34] Wang L., Sun N., Tang H., Sun W. (2019). A Review on Comprehensive Utilization of Red Mud and Prospect Analysis. Minerals.

[35] Silveira N.C.G., Martins M.L.F., Bezerra A.C.S., Araújo F.G.S. (2021). Red Mud from the Aluminium Industry: Production, Characteristics, and Alternative Applications in Construction Materials—A Review. Sustainability.

[36] Milenković A., Smičiklas I., Bundaleski N., Teodoro O.M., Veljović Đ., Vukelić N. (2016). The role of different minerals from red mud assemblage in Co(II) sorption mechanism. Colloids Surf. A Physicochem. Eng. Asp..

[37] Paramguru R.K., Rath P.C., Misra V.N. (2005). Trends in red mud utilization—A review. Min. Process Extr. Met. Rev..

[38] Liu Y., Naidu R. (2013). Hidden values in bauxite residue (red mud): Recovery of metals. Waste Manag..

[39] Liu Q., Zhou Y., Lu J., Zhou Y. (2020). Novel cyclodextrin-based adsorbents for removing pollutants from wastewater: A critical review. Chemosphere.

[40] Anil I., Gunday S.T., Bozkurt A., Alagha O. (2020). Design of crosslinked hydrogels comprising poly (vinylphosphonic acid) and bis [2-(methacryloyloxy) ethyl] phosphate as an efficient adsorbent for wastewater dye removal. Nanomaterials.

[41] Obradovic B. (2020). Guidelines for general adsorption kinetics modeling. Hem. Ind..

[42] Qi Y. (2021). The neutralization and recycling of red mud—A review. J. Phys. Conf. Ser..

[43] Collin G.J., Yun Hin T.Y., Vigneswar K., Gianluca L.P. (2020). Application of modified red mud in environmentally-benign applications: A review. Environ. Eng. Res..

[44] Hu Z.P., Gao Z.M., Liu X., Yuan Z.Y. (2018). High-surface-area activated red mud for efficient removal of methylene blue from wastewater. Adsorpt. Sci. Technol..

[45] Bai Y., Pang Y., Wu Z., Li X., Jing J., Wang H., Zhou Z. (2023). Adsorption of Lead from Water Using MnO_2_-Modified Red Mud: Performance, Mechanism, and Environmental Risk. Water.

[46] Kyrii S., Maletskyi Z., Klymenko N., Ratnaweera H., Mitchenko T., Dontsova T., Kosogina I. (2023). Impact of modification by red mud components on the sorption properties of activated carbon. Appl. Surf. Sci. Adv..

[47] Milenković A.S., Smičiklas I.D., Šljivić-Ivanović M.Z., Živković L.S., Vukelić N.S. (2016). Effect of experimental variables onto Co^2^⁺ and Sr^2^⁺ sorption behavior in red mud-water suspensions. J. Environ. Sci. Health A..

[48] Smiljanic S., Smiciklas I., Peric-Grujic A., Sljivic M., Ðukic B., Loncar B. (2011). Study of factors affecting Ni²⁺ immobilization efficiency by temperature activated red mud. Chem. Eng. J..

[49] Smiljanic S., Smiciklas I., Peric-Grujic A., Loncar B., Mitric M. (2010). Rinsed and thermally treated red mud sorbents for aqueous Ni²⁺ ions. Chem. Eng. J..

[50] Smičiklas I., Smiljanić S., Perić-Grujić A., Šljivić-Ivanović M., Antonović D. (2013). The influence of citrate anion on Ni(II) removal by raw red mud from aluminium industry. Chem. Eng. J..

[51] Smičiklas I., Smiljanić S., Perić-Grujić A., Šljivić-Ivanović M., Mitrić M., Antonović D. (2014). Effect of acid treatment on red mud properties with implications on Ni(II) sorption and stability. Chem. Eng. J..

[52] Šljivić-Ivanović M., Smičiklas I., Dimović S.D., Jović M., Dojčinović B. (2015). Study of Simultaneous Radionuclide Sorption by Mixture Design Methodology. Ind. Eng. Chem. Res..

[53] Sahu M.K., Mandal S., Dash S.S., Badhai P., Patel R.K. (2013). Removal of Pb(II) from aqueous solution by acid activated red mud. J. Environ. Chem. Eng..

[54] Yang T., Wang Y., Sheng L., He C., Sun W., He Q. (2020). Enhancing Cd(II) sorption by red mud with heat treatment: Performance and mechanisms of sorption. J. Environ. Manag..

[55] Lu Z., Qi X., Zhu X., Li X., Li K., Wang H. (2021). Highly effective remediation of high-arsenic wastewater using red mud through formation of AlAsO4@silicate precipitate. Environ. Pollut..

[56] Wu W., Chen D., Li J., Su M., Chen N. (2018). Enhanced adsorption of uranium by modified red muds: Adsorption behavior study. Environ. Sci. Pollut. Res. Int..

[57] Wu W., Chen Z., Huang Y., Li J., Chen D., Chen N., Su M. (2021). Red mud for the efficient adsorption of U(VI) from aqueous solution: Influence of calcination on performance and mechanism. J. Hazard. Mater..

[58] Li C., Yu J., Li W., He Y., Qiu Y., Li P., Wang C., Huang F., Wang D., Gao S. (2018). Immobilization, enrichment and recycling of Cr(VI) from wastewater using a red mud/carbon material to produce the valuable chromite (FeCr_2_O_4_). Chem. Eng. J..

[59] Yang T., Sheng L., Wang Y., Wyckoff K.N., He C., He Q. (2018). Characteristics of Cadmium Sorption by Heat-Activated Red Mud in Aqueous Solution. Sci. Rep..

[60] Lyu F., Niu S., Wang L., Liu R., Sun W., He D. (2021). Efficient removal of Pb(II) ions from aqueous solution by modified red mud. J. Hazard. Mater..

[61] Shabani E., Salimi F., Jahangiri A. (2019). Removal of arsenic and copper from water solution using magnetic iron/bentonite nanoparticles (Fe_3_O_4_/bentonite). Silicon.

[62] Šljivić-Ivanović M., Smičiklas I., Pejanović S. (2013). Analysis and comparison of mass transfer phenomena related to Cu^2+^ sorption by hydroxyapatite and zeolite. Chem. Eng. J..

[63] Milenković A., Smičiklas I., Marković J.P., Vukelić N. (2014). Immobilization of 60Co and 90Sr ions using red mud from aluminium industry. Nucl. Technol. Radiat. Prot..

[64] Smičiklas I., Coha I., Jović M., Nodilo M., Šljivić Ivanović M., Smiljanić S., Grahek Ž. (2021). Efficient separation of strontium radionuclides from high-salinity wastewater by zeolite 4A synthesized from Bayer process liquids. Sci. Rep..

[65] Šljivić-Ivanović M.Z., Smičiklas I.D., Marković J.P., Milenković A. (2013). Analysis of factors influencing Cu(II) sorption by clinoptilolite. Hem. Ind..

[66] Smičiklas I., Dimović S., Šljivić M., Lončar B., Mitrić M. (2010). Resource recovery of animal bones: Study on sorptive properties and mechanism for Sr^2+^ ions. J. Nucl. Mater..

[67] Šljivić-Ivanović M., Milenković A., Jović M., Dimović S., Mraković A., Smičiklas I. (2016). Ni(II) immobilization by bio-apatite materials: Appraisal of chemical, thermal, and combined treatments. Chem. Ind. Chem. Eng. Q..

[68] Šljivić-Ivanović M., Jelić I., Dimović S., Antonijević D., Jović M., Mraković A., Smičiklas I. (2018). Exploring innovative solutions for aged concrete utilization: Treatment of liquid radioactive waste. Clean. Technol. Environ. Policy.

[69] Jelic I., Sljivic-Ivanovic M., Dimovic S., Antonijevic D., Jovic M., Mirkovic M., Smiciklas I. (2018). The Applicability of Construction and Demolition Waste Components for Radionuclide Sorption. J. Clean. Prod..

[70] Jelić I., Antonijević D., Šljivić-Ivanović M., Dimović S. (2023). Application of composite construction and demolition debris in heavy metals removal from industrial wastewater. Therm. Sci..

[71] Jelić I., Šljivić-Ivanović M., Dimović S., Antonijević D., Jović M., Vujović Z., Smičiklas I. (2019). Radionuclide Immobilization by Sorption onto Waste Concrete and Bricks—Experimental Design Methodology. Water Air Soil. Pollut..

[72] Jelić I., Šljivić-Ivanović M., Dimović S., Antonijević D., Jović M., Serović R., Smičiklas I. (2017). Utilization of waste ceramics and roof tiles for radionuclide sorption. Process Saf. Environ. Prot..

[73] Dimović S., Jelić I., Šljivić-Ivanović M., Štirbanović Z., Gardić V., Marković R., Savić A., Zakic D. (2022). Application of Copper Mining Waste in Radionuclide and Heavy Metal Immobilization. Clean. -Soil Air Water.

[74] Sokić K., Milojković N., Dapčević A., Jevtić S., Gasik M. (2024). Novel micro- and nano-composite materials for water purification. Hem. Ind..

[75] Ma Y., Lin C., Jiang Y., Lu W., Si C., Liu Y. (2009). Competitive removal of water-borne copper, zinc and cadmium by a CaCO_3-_dominated red mud. J. Hazard. Mater..

[76] Eid A., Abdel-Aleem G. (2024). A systematic approach for design of distributed wastewater treatment systems. Hem. Ind..

[77] Reddy P.S., Reddy N.G., Serjun V., Mohanty B., Das S.K., Reddy K.R., Rao B.H. (2021). Properties and Assessment of Applications of Red Mud (Bauxite Residue): Current Status and Research Needs. Waste Biomass Valor..

[78] Zeng H., Lyu F., Sun W., Zhang H., Wang L., Wang Y. (2020). Progress on the Industrial Applications of Red Mud with a Focus on China. Minerals.

[79] Gauthier A., Omana B., Amin F., Le Coustumer P. (2024). Waste Bauxite Residue Valorization as Trace Metal Sorbent: Application to Acid Mine Drainage Remediation. Water.

[80] Forghani Tehrani G., Rubinos D.A., Rahimi-Nia A., Bagherian G., Goudarzi N. (2023). Lead(II) removal from aqueous solutions and battery industry wastewater by sorption using seawater-neutralized red mud. Int. J. Environ. Sci. Technol..

[81] Chen Z., Su M., Chen N., Liang D., Chen D. (2022). Effectiveness and mechanism of uranium adsorption on size-graded red mud. Environ. Res..

[82] Bai B., Bai F., Li X., Nie Q., Jia X., Wu H. (2022). The remediation efficiency of heavy metal pollutants in water by industrial red mud particle waste. Environ. Technol. Innov..

[83] Zhao D., Deng H., Wang W., Hu L., Ye S., Fu J., Zhang S. (2025). Synthesis, characterization and adsorption of Pb(II), Cd(II) and Cu(II) by red mud/polyacrylic acid/sodium carboxymethyl cellulose hydrogel. Arab. J. Chem..

[84] Kumar R., Laskar M.A., Hewaidy I.F., Barakat M.A. (2019). Modified Adsorbents for Removal of Heavy Metals from Aqueous Environment: A Review. Earth Syst. Environ..

[85] Wang L., Hu G., Lyu F., Yue T., Tang H., Han H., Yang Y., Liu R., Sun W. (2019). Application of Red Mud in Wastewater Treatment. Minerals.

[86] Li J., Li X., Fischel M., Lin X., Zhou S., Zhang L., Wang L., Yan J. (2024). Applying Red Mud in Cadmium Contamination Remediation: A Scoping Review. Toxics..

[87] Niu A., Lin C. (2024). Trends in research on characterization, treatment and valorization of hazardous red mud: A systematic review. J. Environ. Manag..

